# Comment on Impact of Preoperative Immunonutrition on the Outcomes of Colon Cancer Surgery: Results From a Randomized Controlled Trial

**DOI:** 10.1097/AS9.0000000000000281

**Published:** 2023-04-17

**Authors:** Karem Slim, Cécile Chambrier

**Affiliations:** From the * Department of Digestive Surgery, University Hospital Clermont-Ferrand, France; † Francophone Group for Enhanced Recovery After Surgery (GRACE), Beaumont, France; ‡ Department of Nutrition, Centre Hospitalier Lyon Sud, Hospices Civils de Lyon, Lyon, France.

We read with interest the article published by Lee et al^1^ on the role of immunonutrition (IN) in colon cancer surgery. The authors concluded that routine administration of IN should be abandoned because it was not associated with less infectious complications. However, their trial design is open to criticism. They found no statistically significant difference between the groups for postoperative overall infectious complications (17.7% with IN vs 15.9% without IN; *P* = 0.75). Whenever trial results are negative, a reader’s first reaction is to check whether the sample size has been calculated. Here it was indeed calculated, but wrongly. The authors based their calculation on the results of an earlier Cochrane review (CR) indicating infectious complication rates of 13% with IN and 30% without IN.^2^ In our opinion, this basis is inappropriate because the CR included several gastrointestinal malignancies, without a subgroup analysis of colorectal cancer patients. The CR data cannot, therefore, be extrapolated to the present trial and so cannot be used to calculate the sample size. The Korean data reported in Table 2 show a rate of infectious complications without IN of nearly 16%, markedly <30% rate in the CR (used to calculate the sample size). The authors would be better advised to use their own infectious complications rates to calculate the sample size. Considering their reported rates of infectious complications, many more patients should have been included. The trial is thus clearly underpowered, and so cannot challenge the role of IN either in our daily practice or in the learned societies guidelines. To the best of our knowledge, there is 1 published meta-analysis (including 9 randomized trials) focusing on IN before colorectal surgery.^3^ It showed a beneficial effect of IN (odds ratio, 0.33 [confidence interval, 0.21–0.53]), with no heterogeneity.

In addition, the immunonutrient-enriched supplement used in this study is not really an usual IN. The components were in this study: Arginine 1 g and n-3 fatty acids: 0.92 g while they are: Arginine: 13.3 g and n-3 fatty acids: 3.55 g in the usual IN) suggesting that the amount of immunonutrients was not sufficient to obtain a clinical effect. Furthermore, patients included in this study were not malnourished.

In the era of enhanced recovery programs, the question also arises of whether IN is beneficial in such patients. A preliminary response was provided by the randomized trial published by Moya et al^4^ including 288 patients. This trial clearly suggested that IN was beneficial regarding infectious complications (relative risk, 0.38 [confidence interval, 0.18–0.78]) even in enhanced recovery programs.

To conclude, owing to methodological flaws, the trial published by Lee et al^1^ does not challenge the role of IN in colorectal surgery, although the debate is not over. Until further trials are undertaken, particularly in the setting of well-implemented enhanced recovery programs, the available evidence does not justify giving up preoperative IN in digestive cancer surgery in general. The next question will be how to select the best candidates (ie, frail patients) for IN.
